# Study of Oxygen Saturation by Pulse Oximetry and Arterial Blood Gas in ICU Patients: A Descriptive Cross-sectional Study

**DOI:** 10.31729/jnma.5536

**Published:** 2020-10-31

**Authors:** Nabin Kumar Rauniyar, Shyam Pujari, Pradeep Shrestha

**Affiliations:** 1 Department of Internal Medicine, Nepal Police Hospital, Nepal; 2 Department of Internal Medicine, Dhaulagiri Zonal Hospital, Baglung, Nepal

**Keywords:** *arterial blood oxygen saturation*, *arterial blood gases*, *hypoxemia*, *pulse oximetry*

## Abstract

**Introduction:**

Pulse oximetery is expected to be an indirect estimation of arterial oxygen saturation. However, there often are gaps between SpO2 and SaO2. This study aims to study on arterial oxygen saturation measured by pulse oximetry and arterial blood gas among patients admitted in intensive care unit.

**Methods:**

It was a hospital-based descriptive cross-sectional study in which 101 patients meeting inclusion criteria were studied. SpO2 and SaO2 were measured simultaneously. Mean±SD of SpO2 and SaO2 with accuracy, sensitivity and specificity were measured.

**Results:**

According to SpO2 values, out of 101 patients, 26 (25.7%) were hypoxemic and 75 (74.25%) were non-hypoxemic. The mean±SD of SaO2 and SpO2 were 93.22±7.84% and 92.85±6.33% respectively. In 21 patients with SpO2<90%, the mean±SD SaO2 and SpO2 were 91.63±4.92 and 87.42±2.29 respectively. In 5 patients with SpO2 < 80%, the mean ± SD of SaO2 and SpO2 were: 63.40 ± 3.43 and 71.80±4.28, respectively. In non-hypoxemic group based on SpO2 values, the mean±SD of SpO2 and SaO2 were 95.773±2.19% and 95.654±3.01%, respectively. The agreement rate of SpO2 and SaO2 was 83.2%, and sensitivity and specificity of PO were 84.6% and 83%, respectively.

**Conclusions:**

Pulse Oximetry has high accuracy in estimating oxygen saturation with sp02>90% and can be used instead of arterial blood gas.

## INTRODUCTION

Pulse oximetry (PO) is a useful tool for clinical and investigational purposes for indirect measurements of oxygen saturation.^1–3^ Measurement of oxygen saturation with SpO2 can be used for evaluation and control of hypoxemia. As PO is a non-invasive device, it can be used instead of ABG. Some studies suggest that this method does not exactly reflect the values of ABGs.^2,5–7^.

The majority of patients admitted in ICU show gaps between arterial oxygen saturation measured by ABG and PO. Many studies suggest that pulse oximeters are inaccurate at low saturations^8–12^, because as SaO2 decreases, bias will be increased, while precision (the standard deviation of the differences) will be decreased, with SpO2 increasingly overestimating SaO2.^13^

This study aims to study on arterial oxygen saturation measured by pulse oximetry and arterial blood gas among patients admitted in intensive care unit.

## METHODS

A descriptive cross-sectional study was conducted in ICU of Bir Hospital, Mahaboudha, Kathmandu from Oct 2017 to Oct 2018 after taking ethical clearance from Institutional Review Board, National Academy of Medical Sciences (Ref: 1054). History, clinical examination, chest X-ray findings, pulse oximetry and ABG were reviewed. Patients admitted in ICU with or without hypoxemia during the study period were included in the study after obtaining informed consent.

**Inclusion Criteria**

- Patient older than 18 years admitted in ICU.
- Arterial blood gas analysis and pulse oximetry should be taken simultaneously
- Informed consent is given by the patient (and from the relatives of the patients who were unconscious).

**Exclusion criteria**

- Data were excluded when SaO2 was less than 60% or PaO2 were higher than 100mmHg.
- Patients under 18 years of age.
- Venous blood sample

Convenient sampling was done and the sample size was calculated using the formula,

$$\begin{array}{ll} \text{N} & \text{= }{\text{Z}}^{\text{2}}\times (\text{p}\times \text{q)}/{\text{d}}^{\text{2}}\\ & \text{= }1.96^{\text{2}}\times (94\times 6)/5^{\text{2}}\\ & \text{= }86.6 \end{array}$$

where,

Z = 1.96 ( reliability coefficient at 95 % confidence level)
p = 94 (prevelance of study)
q = 100-p
d = maximum tolerable error = 5
Z = 1.96 at 95 % CI

The calculated minimum sample size was 86.6, however, the total sample size taken was 101. Data were collected using a structured proforma covering the relevant details.

ABG and pulse oximetry were taken simultaneously in all ICU admitted patients Arterial blood sample (about 0. 5ml) was obtained from the radial artery following confirmation of collateral vessel flow by Allen's test or modified Allen test. These are bedside tests that demonstrate collateral flow through the superficial palmar arch.

Before taking the sample, the syringe lumen was heparinized (0.1 ccs). Air bubbles, if present, was immediately expelled from the sample; the sample was sealed in an iced container and taken to blood gas analyzer (ABL 80Flex Automatic Blood Gases, Copenhagen, Denmark).

SpO2 was obtained using a pulse oximeter (Fingertip, China). The finger probe for the unit was placed on the index finger of the opposite arm from which the arterial sample had been taken. Spo2 was measured twice at 0 minutes and 2 minutes, then the average of two was taken. In this study, ABG values were taken as reference values. According to SpO2, the studied patients were divided into three groups: SpO2 <80%, 90%>Sp02≥80% and SpO2 ≥ 90%. Hypoxemia was considered as SpO2 or SaO2 <90% or partial pressure of oxygen (Pa02) < 60mm Hg by ABG.

The data were collected and entered in MS-Excel 2007 and analyzed using the IBM Statistical Package for Social Sciences (SPSS) version 20 software. Sensitivity, specificity and accuracy of PO were measured and calculated.

## RESULTS

A total of 101 patients were enrolled in this study (56 Male, 45 Female). Twenty six (25.7%) were hypoxemic and 75 (74.25%) were non-hypoxemic according to PO and based on ABG results, SaO2 values were less than 90% in 13 (12.9%) patients, and the values were equal or more than 90% in 88 (87.1%) patients. The mean ± SD oxygen saturation values (SaO2) measured by ABG analyzer system were greater than those measured by pulse oximetry (SpO2): (SaO2 = 93.22±7.84%, SpO2 = 92.85±6.33%).

There were differences between pulse oximetry (SpO2) and ABG values (SaO2) in the limit of SpO2<80% and 90% >SpO2 ≥80% (Table 1).

**Table 1:** Statistical indexes of oxygen saturation values measured by ABG and PO for arterial oxygen saturation.

Statistical Analysis		Oxygen saturation (mean ± SD)
Group	Methods	
Overall	ABG	93.22±7.84%
(n = 101)	Pulse Oximetry	92.85±6.33%
SpO2 < 80%	ABG	63.40 ± 3.43511
(n = 5)	Pulse Oximetry	71.80 ± 4.280
90% > SpO2 ≥80%	ABG	91.63±4.92
(n = 21)	Pulse Oximetry	87.42±2.293
SpO2 ≥ 90%	ABG	95.6547±3.013
(n = 75)	Pulse Oximetry	95.77±2.192

In 21 hypoxemic patients (SpO2<90%), the mean ± SD SaO2 and SpO2 were: 91.63±4.92 and 87.42±2.29. In 5 of the hypoxemic patients (SpO2 < 80%), the mean ± SD of SaO2 and SpO2 were: 63.40±3.43 and 71.80±4.28, respectively. As patients were defined non-hypoxemic based on SpO2 values, the mean ± SD of SpO2 and SaO2 were 95.773±2.19%, 95.654±3.01%, respectively. The sensitivity and specificity of pulse oximetry were 84.6 % and 83 %, respectively (Table 2).

**Table 2:** Sensitivity, specificity and accuracy of the Pulse oxymetry.

		Arterial Blood Gas (ABG)	
		Hypoxic (<90)	Non-hypoxic (90 or more)
Pulse oxymetry	Hypoxic (<90)	11 (84.6%)	15 (17%)
	Non-hypoxic (90 or more)	2 (15.4%)	73 (83%)

## DISCUSSION

In acute illness, patients with SpO2 ≥ 90%, PO has high accuracy in estimating SaO2 and may be used instead of ABG. The study suggests that in patients with SpO2 < 90%, however, the exact estimation of SaO2 and the evaluation of oxygenation by pulse oximeter is not a good substitution for ABG analyzer.

Regarding the comparison between oxygen saturation measured by pulse oximetry and ABG, many studies have been conducted related to accuracy.

In a recent meta-analysis of the measurement of SaO2 by pulse oximetry, Jensen et al. concluded that, from the 74 studies (1976 to 1994 ), pulse oximeters were accurate within 2% in the range of 70-100% SaO2.^14^ In the study of Carter et al., the performance of pulse oximetry deteriorated below a SpO2 of 75%.^15^ In the study of Chiappini et al., a significant difference was found between SpO2 and SaO2 values.^16^ SpO2 values were lower than SaO2 (90.58±5.45% vs. 92.14±5.79%) and a lack of accuracy of the pulse oximeter was found, but only for SpO2 values below 82%.

Study of Kenneth P Levin et al conducted in 2001, shows that increasing the assessment of arterial oxygenation among patients with community-acquired pneumonia(CAP) is likely to increase the detection of arterial hypoxemia, particularly among outpatients. However, ABG should be considered for patients with underlying cardiopulmonary disease, for those for whom ventilatory failure is of clinical concern, and if other reported sources of error for PO are present.^17^

Similarly, another study of Ebrahim Razi, Hossein Akbari in 2006 done for COPD patients included 152 patients.^18^ The accuracy of pulse oximetry was 90.8%, sensitivity was 93.3% and specificity was 89.1%. The study reported that in pulmonary diseases with SpO2 ≥ 80%, PO has high accuracy in estimating SaO2, which is contradictory to our study where accuracy is estimated when Spo2 > 90%. In patients with SpO2 < 80%, however, the result is similar to our study and concluded that the evaluation of oxygenation by pulse oximeter is not a good substitution for ABG analyzer.

In a study of Kanai R., Moriyama K. et al done in large scale including 20717 arterial blood gas samples from ICU patients, SpO2 tended to show a higher value than SaO2 and suggest to keep SpO2 above 92% to avoid hypoxemia in the ICU to decrease morbidity and mortality.^19^

Webb et al. reported that pulse oximetry is poorly calibrated at low saturations and generally less accurate and less precise than at normal saturations.^20^ In the study of Webb et al., nearly 30% of values reviewed were erroneous by more than 5% at saturation of less than 80%.

Many studies suggest that pulse oximeters are inaccurate at low saturations^8–12^, because as SaO2 decreases, bias will be increased, while precision (the standard deviation of the differences) will be decreased, with SpO2 increasingly overestimating SaO2.^13^ Many explanations have been proposed for the limited performance of pulse oximeters at low saturations. One is the slight variations in the output wavelength of the light-emitting diodes which generate proportionally larger errors at low saturations.^21,22^ Another is the generation of proportionally larger errors in the measurement of transmitted red light versus of infra-red light at low saturations because of the large extinction coefficient of reduced haemoglobin.^23^

In the current study when oxygenation saturation was more than 90%, there was good correlation coefficient between the two methods of measurements (in non-hypoxemic groups, the correlation coefficient was 0.493 and P<0.001). The study revealed that only two patients (15.4%) out of all who were considered non-hypoxemic according to PO were considered hypoxemic in terms of ABG values. The obtained agreement rate was 83.2%. Given the critical point of Sp02≥90%, about 83.2% of cases correlated this regard. Thus, although patients were considered as hypoxemic or non-hypoxemic according to pulse oximetry or ABG results, because of slight changes among the results of the two methods and also a wide variety of cases, correlation coefficient had shown good correlation between two methods, especially in Spo2≥90% with P<0.001.

From the above discussion, we can conclude that PO is easily available, painless, non-invasive continuous monitoring and cheap method that can be considered an appropriate substitute for ABG, especially in SpO2 ≥90% with an accuracy of about 83.2%. However in conditions with low oxygen saturation (SpO2 <90%) and in critical status, PO is a poor predictor of hypoxemia or not a good alternative to estimate arterial oxygen saturation. There are several limitations of the study as the sample size is small and the accuracy rate is only 83.2%. Futher studies are required for accuracy and effectiveness of the PO in a large sample. In addition, though PO measures arterial oxygenation, it does not assess ventilation. Therefore, it needs to be aided with ABG analysis when alveolar hypoventilation is suspected clinically.

## CONCLUSIONS

Pulse Oximetry has high accuracy in estimating oxygen saturation with sp02>90% and can be used instead of arterial blood gas. The study suggests that in patients with SpO2 <90%, however, the exact estimation of SaO2 and the evaluation of hypoxemia by pulse oximeter is not a good alternative for ABG analyzer.
